# 5-Fluoro-1-[(4*S*,5*R*)-5-(2-hydroxy­ethyl)-2,2-dimethyl-1,3-dioxolan-4-yl]pyrimidine-2,4(1*H*,3*H*)-dione

**DOI:** 10.1107/S1600536810016065

**Published:** 2010-05-12

**Authors:** Angel Mendoza, Martha Sosa-Rivadeneyra, Fernando Sartillo-Piscil, Leticia Quintero, Marcos Flores-Alamo

**Affiliations:** a Centro de Química, ICUAP, Benemérita Universidad Autónoma de Puebla, Puebla, Pue., Mexico; b Facultad de Ciencias Químicas, Benemérita Universidad Autónoma de Puebla, Puebla, Pue., Mexico; c Facultad de Química, Universidad Nacional Autónoma de México, 04510, México, DF, Mexico

## Abstract

In the title compound, C_11_H_15_FN_2_O_5_, the five-membered ring has an envelope conformation, while the six-membered ring is essentially planar, with a maximum deviation of 0.032 (2) Å from the mean plane. The crystal packing is stabilized by inter­molecular N—H⋯O and O—H⋯O hydrogen bonds, generating a layer structure parallel to (001).

## Related literature

For applications of modified nucleosides in medical chemistry, see: Huryn & Okabe (1992 ▶); Minuk *et al.* (1992 ▶); Luscombe *et al.* (1996 ▶); Korba & Boyd (1996 ▶). For the synthesis, see: Valdivia *et al.* (2005 ▶); Xie *et al.* (1996 ▶). For ring conformation analysis, see: Cremer & Pople (1975 ▶).
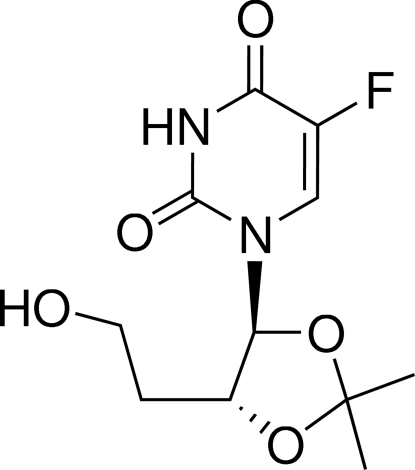


## Experimental

### 

#### Crystal data

C_11_H_15_FN_2_O_5_
*M* *_r_* = 274.25
Monoclinic, 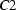

*a* = 20.8905 (8) Å
*b* = 5.5751 (1) Å
*c* = 13.5639 (5) Å
β = 126.297 (6)°
*V* = 1273.21 (12) Å^3^
*Z* = 4
Cu *K*α radiation
μ = 1.06 mm^−1^
*T* = 298 K
0.40 × 0.12 × 0.08 mm

#### Data collection

Oxford Diffraction Gemini Atlas CCD diffractometer
Absorption correction: analytical (*CrysAlis RED*; Oxford Diffraction, 2009 ▶) *T*_min_ = 0.885, *T*_max_ = 0.964
4606 measured reflections
1786 independent reflections
1732 reflections with *I* > 2σ(*I*)
*R*_int_ = 0.013

#### Refinement

*R*[*F* ^2^ > 2σ(*F* ^2^)] = 0.027
*wR*(*F* ^2^) = 0.071
*S* = 1.04
1786 reflections
181 parameters
1 restraint
H atoms treated by a mixture of independent and constrained refinement
Δρ_max_ = 0.23 e Å^−3^
Δρ_min_ = −0.20 e Å^−3^
Absolute structure: Flack (1983 ▶), 498 Friedel pairs
Flack parameter: 0.0 (2)

### 

Data collection: *CrysAlis CCD* (Oxford Diffraction, 2009 ▶); cell refinement: *CrysAlis RED* (Oxford Diffraction, 2009 ▶); data reduction: *CrysAlis RED*; program(s) used to solve structure: *SHELXS97* (Sheldrick, 2008 ▶); program(s) used to refine structure: *SHELXL97* (Sheldrick, 2008 ▶); molecular graphics: *ORTEP-3 for Windows* (Farrugia, 1997 ▶); software used to prepare material for publication: *WinGX* (Farrugia, 1999 ▶).

## Supplementary Material

Crystal structure: contains datablocks I, global. DOI: 10.1107/S1600536810016065/is2543sup1.cif

Structure factors: contains datablocks I. DOI: 10.1107/S1600536810016065/is2543Isup2.hkl

Additional supplementary materials:  crystallographic information; 3D view; checkCIF report

## Figures and Tables

**Table 1:** Hydrogen-bond geometry (Å, °)

*D*—H⋯*A*	*D*—H	H⋯*A*	*D*⋯*A*	*D*—H⋯*A*
N2—H1*N*⋯O5^i^	0.83 (2)	2.01 (2)	2.828 (2)	167 (3)
O5—H1*O*⋯O2^ii^	0.75 (3)	2.16 (3)	2.876 (2)	160 (3)

Symmetry codes: (i) 
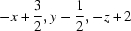
; (ii) 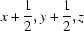.

**Fig. 1.**
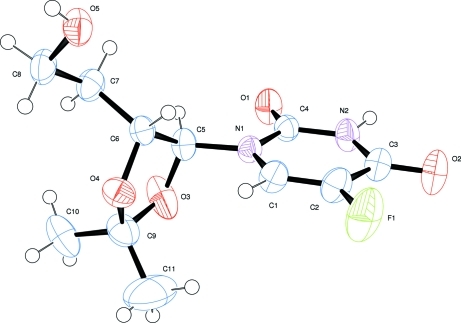
The molecular structure of compound 1, with atom labels and 50% probability displacement ellipsoids for non-H atoms.

**Fig. 2.**
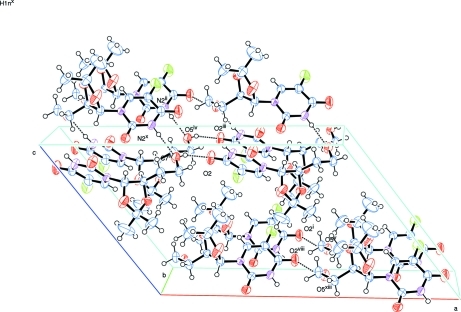
The packing of compound 1, viewed down the b axis, showing one layer of molecules connected by O5—H···O2 and N2—H···O5 hydrogen bonds (dashed lines).

## supplementary crystallographic information

### Comment

For many years, design of modified nucleosides has been a focal point of research in medicinal chemistry (Huryn & Okabe, 1992). Modified nucleosides have acquired an important role as therapeutic agents for the treatment of patients with devastating infections with viruses such as human immunodeficiency virus (HIV), hepatitis B virus (HBV), and herpes viruses. A class of nucleoside analogues for antiviral chemotherapy is that where cyclic carbohydrate moiety is replaced with open-chain "acyclic" sugar moieties. Among purine acyclic nucleosides, are Acyclovir, Ganciclovir and Penciclovir (Minuk *et al.*, 1992; Luscombe *et al.*, 1996; Korba & Boyd, 1996).

In this context and as result of our continuing investigations on the synthesis of nucleoside analogues, we report a new compound 1 (Scheme 1). This new analogue might present a similarity with a number of acyclic nucleosides, which showed remarkable antiviral properties.

In the present paper, we report the structure of title compound 1. In the [(1'*S*, 2'*R*)-(1', 2'-*O*-isopropylidene-4'-hydroxy-1-butyl)], the five member ring (C5/C6/O3/O4/C9) shows an envelope conformation on atom C6 with puckering parameters (Cremer & Pople, 1975) *q*_2_ = 0.238 (2) Å and φ_2_ = 69.8 (6)°. For the six member ring uracil, shows a planar configuration with torsion angle (N1—C4—N2—C3) of 4.8 (3)°, and C1—C2 = 1.325 (3) Å and N2—C4 = 1.384 (2) Å (double bond). The crystal packing is stabilized by two intermolecular hydrogen bonds [O5···O2 = 2.828 (2) Å and N2···O5 = 2.876 (2) Å], generating a layer parallel to the (001) plane.

### Experimental

Deshomologation of previos nucleoside analogue, 1-[(1'*S*,2'*R*,4'*S*)-(1',2'-*O*-isopropylidene-4',5'-dihydroxy-1'-pentyl)]-5-fluorouracil, was achieved following non-aqueous protocol (Valdivia *et al.*, 2005; Xie *et al.*, 1996). Reaction was carried out by two steps: a) periodic acid/ethyl acetate, 30 min, b) EtOH/H2O/ NaBH4, 20 min, rt. Final purification of compound 1 was achieved by crystallization from hexane. Yield 80%, white solid, m.p. 184 °C; [α]_D_ -18.51 (c 1.0, CH_3_OH). ^1^H NMR (300 MHz, CDCl_3_/TMS) 1.53 (s, 3H), 1.57 (s, 3H), 1.95 (m, 1H), 2.11 (m, 1H), 3.80 (m, 2H), 4.21 (m, 1H), 5.90 (d, 1H, J = 5.1 Hz), 7.3 (s, 1H). ^13^C MNR (75 MHz, CDCl_3_/TMS) 26.9, 27.9, 34.9, 55.8, 58.9, 79.4, 86.8, 111.6, 142.2, 149.5, 157.5.

### Refinement

H atoms bonded to N2 and O5 atoms were located in a difference Fourier map and refined with free coordinates and isotropic *U* parameters. H atoms linked to C atoms were placed in geometrical idealized positions and refined as riding on their parent atoms, with C—H = 0.93–0.98 Å, and with *U*_iso_(H) = 1.2*U*_eq_(C) or 1.5*U*_eq_(C) for methyl groups.

### Crystal data

**Table:**

C_11_H_15_FN_2_O_5_	*F*(000) = 576
*M**_r_* = 274.25	*D*_x_ = 1.431 Mg m^−^^3^
Monoclinic, *C*2	Cu *K*α radiation, λ = 1.54184 Å
Hall symbol: C 2y	Cell parameters from 4215 reflections
*a* = 20.8905 (8) Å	θ = 4.0–68.0°
*b* = 5.5751 (1) Å	µ = 1.06 mm^−^^1^
*c* = 13.5639 (5) Å	*T* = 298 K
β = 126.297 (6)°	Prism, colorless
*V* = 1273.21 (12) Å^3^	0.40 × 0.12 × 0.08 mm
*Z* = 4	

### Data collection

**Table:**

Oxford Diffraction Gemini Atlas CCD diffractometer	1786 independent reflections
Radiation source: fine-focus sealed tube	1732 reflections with *I* > 2σ(*I*)
graphite	*R*_int_ = 0.013
Detector resolution: 10.4685 pixels mm^-1^	θ_max_ = 68.1°, θ_min_ = 4.0°
ω scans	*h* = −22→24
Absorption correction: analytical (*CrysAlis RED*; Oxford Diffraction, 2009)	*k* = −4→6
*T*_min_ = 0.885, *T*_max_ = 0.964	*l* = −16→16
4606 measured reflections	

### Refinement

**Table:**

Refinement on *F*^2^	Hydrogen site location: inferred from neighbouring sites
Least-squares matrix: full	H atoms treated by a mixture of independent and constrained refinement
*R*[*F*^2^ > 2σ(*F*^2^)] = 0.027	*w* = 1/[σ^2^(*F*_o_^2^) + (0.038*P*)^2^ + 0.4768*P*] where *P* = (*F*_o_^2^ + 2*F*_c_^2^)/3
*wR*(*F*^2^) = 0.071	(Δ/σ)_max_ < 0.001
*S* = 1.04	Δρ_max_ = 0.23 e Å^−^^3^
1786 reflections	Δρ_min_ = −0.19 e Å^−^^3^
181 parameters	Extinction correction: *SHELXL97* (Sheldrick, 2008), Fc^*^=kFc[1+0.001xFc^2^λ^3^/sin(2θ)]^-1/4^
1 restraint	Extinction coefficient: 0.0075 (4)
Primary atom site location: structure-invariant direct methods	Absolute structure: Flack (1983), 498 Friedel pairs
Secondary atom site location: difference Fourier map	Flack parameter: 0.0 (2)

### Special details

**Table:**

Geometry. All s.u.'s (except the s.u. in the dihedral angle between two l.s. planes) are estimated using the full covariance matrix. The cell s.u.'s are taken into account individually in the estimation of s.u.'s in distances, angles and torsion angles; correlations between s.u.'s in cell parameters are only used when they are defined by crystal symmetry. An approximate (isotropic) treatment of cell s.u.'s is used for estimating s.u.'s involving l.s. planes.
Refinement. Refinement of *F*^2^ against ALL reflections. The weighted *R*-factor wR and goodness of fit *S* are based on *F*^2^, conventional *R*-factors *R* are based on *F*, with *F* set to zero for negative *F*^2^. The threshold expression of *F*^2^ > 2σ(*F*^2^) is used only for calculating *R*-factors(gt) etc. and is not relevant to the choice of reflections for refinement. *R*-factors based on *F*^2^ are statistically about twice as large as those based on *F*, and *R*- factors based on ALL data will be even larger.

### Fractional atomic coordinates and isotropic or equivalent isotropic displacement parameters (Å^2^)

**Table:**

	*x*	*y*	*z*	*U*_iso_*/*U*_eq_	
O1	0.69200 (8)	0.0051 (3)	0.95722 (12)	0.0519 (4)	
N1	0.66380 (8)	0.2920 (3)	0.81679 (12)	0.0339 (3)	
O4	0.72382 (7)	0.3751 (3)	0.65042 (11)	0.0438 (3)	
F1	0.52029 (9)	0.7673 (3)	0.69645 (15)	0.0816 (5)	
O5	0.90402 (9)	0.7069 (3)	0.88915 (13)	0.0478 (4)	
O2	0.49424 (9)	0.5250 (3)	0.84757 (15)	0.0627 (5)	
C5	0.72230 (11)	0.1894 (4)	0.80156 (17)	0.0403 (4)	
H5	0.7609	0.0950	0.8745	0.048*	
N2	0.59707 (9)	0.2801 (4)	0.90550 (15)	0.0434 (4)	
C1	0.61955 (10)	0.4901 (4)	0.75175 (16)	0.0418 (4)	
H1	0.6291	0.5673	0.7009	0.050*	
O3	0.68552 (11)	0.0402 (3)	0.69943 (16)	0.0681 (5)	
C4	0.65428 (10)	0.1784 (4)	0.89752 (15)	0.0362 (4)	
C6	0.76688 (9)	0.3754 (4)	0.78102 (14)	0.0350 (4)	
H6	0.7625	0.5332	0.8084	0.042*	
C2	0.56349 (11)	0.5732 (4)	0.76007 (18)	0.0466 (5)	
C3	0.54650 (10)	0.4653 (4)	0.83818 (17)	0.0435 (5)	
C8	0.89973 (11)	0.4915 (4)	0.83002 (18)	0.0469 (5)	
H8A	0.9528	0.4318	0.8649	0.056*	
H8B	0.8733	0.5206	0.7435	0.056*	
C7	0.85368 (10)	0.3099 (4)	0.84748 (17)	0.0437 (5)	
H7A	0.8779	0.2948	0.9342	0.052*	
H7B	0.8572	0.1551	0.8184	0.052*	
C9	0.68429 (13)	0.1521 (4)	0.60232 (19)	0.0533 (6)	
C10	0.72705 (17)	−0.0147 (5)	0.5721 (2)	0.0689 (7)	
H10A	0.6979	−0.1621	0.5400	0.103*	
H10B	0.7793	−0.0473	0.6448	0.103*	
H10C	0.7313	0.0588	0.5121	0.103*	
C11	0.60061 (17)	0.2034 (8)	0.4934 (3)	0.1094 (13)	
H11A	0.5725	0.0550	0.4592	0.164*	
H11B	0.6016	0.2893	0.4330	0.164*	
H11C	0.5743	0.2986	0.5185	0.164*	
H1N	0.5950 (12)	0.236 (5)	0.9622 (19)	0.048 (6)*	
H1O	0.9348 (15)	0.789 (6)	0.896 (2)	0.072 (9)*	

### Atomic displacement parameters (Å^2^)

**Table:**

	*U*^11^	*U*^22^	*U*^33^	*U*^12^	*U*^13^	*U*^23^
O1	0.0606 (7)	0.0506 (9)	0.0586 (7)	0.0216 (7)	0.0430 (7)	0.0239 (8)
N1	0.0336 (6)	0.0365 (9)	0.0345 (7)	0.0036 (7)	0.0218 (6)	0.0044 (6)
O4	0.0520 (7)	0.0397 (8)	0.0390 (6)	−0.0028 (6)	0.0265 (5)	0.0009 (6)
F1	0.0799 (8)	0.0748 (11)	0.1109 (11)	0.0463 (8)	0.0678 (8)	0.0532 (10)
O5	0.0529 (8)	0.0445 (10)	0.0622 (8)	−0.0061 (7)	0.0430 (7)	−0.0071 (7)
O2	0.0621 (8)	0.0584 (11)	0.0954 (11)	0.0167 (8)	0.0619 (8)	0.0137 (10)
C5	0.0465 (9)	0.0352 (11)	0.0509 (9)	0.0048 (9)	0.0353 (8)	0.0038 (9)
N2	0.0502 (8)	0.0455 (10)	0.0498 (8)	0.0083 (8)	0.0381 (7)	0.0092 (8)
C1	0.0410 (8)	0.0437 (12)	0.0447 (9)	0.0064 (9)	0.0275 (7)	0.0138 (9)
O3	0.1087 (12)	0.0487 (10)	0.0938 (11)	−0.0334 (9)	0.0857 (10)	−0.0292 (9)
C4	0.0368 (8)	0.0390 (11)	0.0359 (8)	0.0011 (8)	0.0232 (7)	0.0018 (8)
C6	0.0397 (8)	0.0320 (10)	0.0388 (8)	0.0022 (8)	0.0263 (7)	0.0006 (8)
C2	0.0444 (9)	0.0382 (12)	0.0563 (11)	0.0122 (9)	0.0294 (9)	0.0139 (9)
C3	0.0417 (8)	0.0401 (12)	0.0557 (10)	0.0025 (9)	0.0326 (8)	0.0013 (9)
C8	0.0430 (9)	0.0492 (13)	0.0608 (11)	0.0008 (10)	0.0374 (9)	−0.0079 (11)
C7	0.0400 (9)	0.0402 (12)	0.0531 (10)	0.0056 (9)	0.0288 (8)	0.0001 (9)
C9	0.0641 (12)	0.0483 (14)	0.0551 (11)	−0.0145 (11)	0.0394 (10)	−0.0142 (10)
C10	0.1132 (18)	0.0523 (16)	0.0761 (14)	−0.0072 (15)	0.0752 (15)	−0.0113 (13)
C11	0.0695 (16)	0.110 (3)	0.086 (2)	−0.0123 (19)	0.0121 (15)	−0.043 (2)

### Geometric parameters (Å, °)

**Table:**

O1—C4	1.206 (2)	O3—C9	1.444 (3)
N1—C1	1.373 (3)	C6—C7	1.516 (2)
N1—C4	1.378 (2)	C6—H6	0.9800
N1—C5	1.471 (2)	C2—C3	1.434 (3)
O4—C9	1.419 (3)	C8—C7	1.509 (3)
O4—C6	1.435 (2)	C8—H8A	0.9700
F1—C2	1.345 (2)	C8—H8B	0.9700
O5—C8	1.417 (3)	C7—H7A	0.9700
O5—H1O	0.75 (3)	C7—H7B	0.9700
O2—C3	1.218 (2)	C9—C11	1.504 (4)
C5—O3	1.393 (3)	C9—C10	1.505 (3)
C5—C6	1.526 (3)	C10—H10A	0.9600
C5—H5	0.9800	C10—H10B	0.9600
N2—C3	1.369 (3)	C10—H10C	0.9600
N2—C4	1.384 (2)	C11—H11A	0.9600
N2—H1N	0.83 (2)	C11—H11B	0.9600
C1—C2	1.325 (3)	C11—H11C	0.9600
C1—H1	0.9300		
			
C1—N1—C4	121.43 (14)	N2—C3—C2	112.25 (16)
C1—N1—C5	121.47 (14)	O5—C8—C7	108.20 (15)
C4—N1—C5	117.10 (15)	O5—C8—H8A	110.1
C9—O4—C6	109.72 (14)	C7—C8—H8A	110.1
C8—O5—H1O	110 (2)	O5—C8—H8B	110.1
O3—C5—N1	110.92 (16)	C7—C8—H8B	110.1
O3—C5—C6	105.14 (14)	H8A—C8—H8B	108.4
N1—C5—C6	114.14 (16)	C8—C7—C6	113.23 (17)
O3—C5—H5	108.8	C8—C7—H7A	108.9
N1—C5—H5	108.8	C6—C7—H7A	108.9
C6—C5—H5	108.8	C8—C7—H7B	108.9
C3—N2—C4	128.17 (16)	C6—C7—H7B	108.9
C3—N2—H1N	113.4 (16)	H7A—C7—H7B	107.7
C4—N2—H1N	118.1 (16)	O4—C9—O3	105.54 (16)
C2—C1—N1	121.28 (17)	O4—C9—C11	107.9 (2)
C2—C1—H1	119.4	O3—C9—C11	111.2 (2)
N1—C1—H1	119.4	O4—C9—C10	112.88 (19)
C5—O3—C9	110.51 (16)	O3—C9—C10	106.8 (2)
O1—C4—N1	123.91 (16)	C11—C9—C10	112.5 (2)
O1—C4—N2	121.80 (16)	C9—C10—H10A	109.5
N1—C4—N2	114.29 (16)	C9—C10—H10B	109.5
O4—C6—C7	112.88 (14)	H10A—C10—H10B	109.5
O4—C6—C5	102.72 (13)	C9—C10—H10C	109.5
C7—C6—C5	111.86 (15)	H10A—C10—H10C	109.5
O4—C6—H6	109.7	H10B—C10—H10C	109.5
C7—C6—H6	109.7	C9—C11—H11A	109.5
C5—C6—H6	109.7	C9—C11—H11B	109.5
C1—C2—F1	121.20 (18)	H11A—C11—H11B	109.5
C1—C2—C3	122.28 (19)	C9—C11—H11C	109.5
F1—C2—C3	116.51 (17)	H11A—C11—H11C	109.5
O2—C3—N2	121.46 (18)	H11B—C11—H11C	109.5
O2—C3—C2	126.3 (2)		
			
C1—N1—C5—O3	−82.0 (2)	N1—C5—C6—C7	141.06 (16)
C4—N1—C5—O3	96.83 (19)	N1—C1—C2—F1	179.91 (19)
C1—N1—C5—C6	36.5 (2)	N1—C1—C2—C3	1.0 (3)
C4—N1—C5—C6	−144.63 (16)	C4—N2—C3—O2	173.9 (2)
C4—N1—C1—C2	−3.0 (3)	C4—N2—C3—C2	−6.5 (3)
C5—N1—C1—C2	175.8 (2)	C1—C2—C3—O2	−177.1 (2)
N1—C5—O3—C9	107.78 (18)	F1—C2—C3—O2	4.0 (3)
C6—C5—O3—C9	−16.1 (2)	C1—C2—C3—N2	3.3 (3)
C1—N1—C4—O1	−179.53 (18)	F1—C2—C3—N2	−175.64 (18)
C5—N1—C4—O1	1.6 (3)	O5—C8—C7—C6	67.2 (2)
C1—N1—C4—N2	0.4 (3)	O4—C6—C7—C8	65.3 (2)
C5—N1—C4—N2	−178.50 (15)	C5—C6—C7—C8	−179.47 (16)
C3—N2—C4—O1	−175.3 (2)	C6—O4—C9—O3	15.2 (2)
C3—N2—C4—N1	4.8 (3)	C6—O4—C9—C11	134.1 (2)
C9—O4—C6—C7	96.42 (19)	C6—O4—C9—C10	−101.1 (2)
C9—O4—C6—C5	−24.21 (19)	C5—O3—C9—O4	1.4 (2)
O3—C5—C6—O4	24.16 (19)	C5—O3—C9—C11	−115.3 (2)
N1—C5—C6—O4	−97.61 (16)	C5—O3—C9—C10	121.8 (2)
O3—C5—C6—C7	−97.17 (18)		

### Hydrogen-bond geometry (Å, °)

**Table:**

*D*—H···*A*	*D*—H	H···*A*	*D*···*A*	*D*—H···*A*
N2—H1N···O5^i^	0.83 (2)	2.01 (2)	2.828 (2)	167 (3)
O5—H1O···O2^ii^	0.75 (3)	2.16 (3)	2.876 (2)	160 (3)

Symmetry codes: (i) −*x*+3/2, *y*−1/2, −*z*+2; (ii) *x*+1/2, *y*+1/2, *z*.
